# Element- and enantiomer-selective visualization of molecular motion in real-time

**DOI:** 10.1038/s41467-023-36047-5

**Published:** 2023-01-24

**Authors:** R. Mincigrucci, J. R. Rouxel, B. Rossi, E. Principi, C. Bottari, S. Catalini, J. S. Pelli-Cresi, D. Fainozzi, L. Foglia, A. Simoncig, A. Matruglio, G. Kurdi, F. Capotondi, E. Pedersoli, A. Perucchi, F. Piccirilli, A. Gessini, M. Giarola, G. Mariotto, M. Oppermann, S. Mukamel, F. Bencivenga, M. Chergui, C. Masciovecchio

**Affiliations:** 1 Elettra Sincrotrone Trieste S.C.p.A., Strada Statale 14 - km 163, 5 in AREA Science Park, 34149 Basovizza, Trieste Italy; 2 Lausanne Centre for Ultrafast Spectroscopy (LACUS), École Polytechnique Fédérale de Lausanne, CH-1015 Lausanne, Switzerland; 3 Univ de Lyon, UJM-Saint-Etienne, CNRS, IOGS, Laboratoire Hubert Curien UMR 5516, Saint-Etienne, F-42023 France; 4 Department of Physics, University of Trento, Via Sommarive 14, 38123 Povo, Trento Italy; 5 Department of Physics, University of Trieste, Trieste, Italy; 6 European Laboratory for Non-Linear Spectroscopy (LENS), Università di Firenze, 50121 Florence, Italy; 7 Department of Physics and Geology, University of Perugia, 06123 Perugia, Italy; 8 CNR-INO, Consiglio Nazionale Delle Ricerche, Istituto Nazionale di Ottica, Largo Fermi 6, 50125 Florence, Italy; 9 CERIC-ERIC, Strada Statale 14 - km 1635 in AREA Science Park, 34149 Basovizza, Trieste Italy; 10 Centro Piattaforme Tecnologiche, University of Verona, Policlinico GB Rossi, Ple. L.A. Scuro, 10, 37134 Verona, Italy; 11 Department of Computer Science, University of Verona, Strada le Grazie 15, 37134 Verona, Italy; 12 Department of Chemistry, University of Basel, Klingelbergstrasse 80, 4056 Basel, Switzerland; 13 Department of Chemistry and physics and astronomy, University of California Irvine, Irvine, CA 92697 USA

**Keywords:** Physical chemistry, Optical techniques

## Abstract

Ultrafast optical-domain spectroscopies allow to monitor in real time the motion of nuclei in molecules. Achieving element-selectivity had to await the advent of time resolved X-ray spectroscopy, which is now commonly carried at X-ray free electron lasers. However, detecting light element that are commonly encountered in organic molecules, remained elusive due to the need to work under vacuum. Here, we present an impulsive stimulated Raman scattering (ISRS) pump/carbon K-edge absorption probe investigation, which allowed observation of the low-frequency vibrational modes involving specific selected carbon atoms in the Ibuprofen RS dimer. Remarkably, by controlling the probe light polarization we can preferentially access the enantiomer of the dimer to which the carbon atoms belong.

## Abstract (web summary)

Complex molecules show element- and enantio-specific properties and reactivity. Here the authors demonstrate identification of the element- and enantiomer-selective motion of Ibuprofen molecule using X-ray photons at the carbon K-edge.

## Introduction

The ability to monitor in real-time the motion of nuclei within a molecule, whether it is diatomic or a macromolecule, became possible during the 1980s with the advent of ultrafast spectroscopy^1^. However, optical spectroscopy is not an element-specific tool, yet the ultimate goal of physical (bio)chemists is to monitor the role of each atom in the course of a (bio)chemical reaction. This led to the development and implementation of time-resolved X-ray^2,3^ and electron diffraction^4,5^, and of X-ray spectroscopy^2,6^. Femtosecond X-ray diffraction and X-ray absorption spectroscopy can map the time evolution of specific atomic elements but this approach was initially limited to systems containing heavy atoms because they efficiently scatter and/or absorb hard X-rays^6–8^. Hard X-ray absorption spectroscopy allows to observe the local atomic environment of a given element as well as its electronic structure. In time-domain experiments, it was used to probe the dynamics of photoexcited metal molecular complexes^2,6^, biomolecules^9,10^ and solids^2^. Extending these methods to the soft X-ray domain, opens an observation window into light elements, such as C, N, O, S, F, etc, and transition metals via their M-edges, which play a crucial role in organic chemistry and biochemistry. This was recently achieved using both table-top set-ups^11^ based on High Harmonic Generation (HHG)^12,13^ and at large scale facilities^14–17^ and X-ray free electron lasers (FEL)^18–20^.

Chiral molecules exist in two different forms, called left and right-handed enantiomers, that are chemically identical but are mirror images of each other. Chirality plays a crucial role in stereochemistry, inorganic chemistry and in biochemistry. Nature is homo-chiral and therefore distinguishing molecular enantiomers is a central issue in pharmacology, toxicology and drug design. The method commonly used to detect enantiomers is circular dichroism (CD) spectroscopy. It exploits the fact that light polarized into a circular wave is absorbed differently by left-handed and right-handed enantiomers. In the spirit of monitoring the evolution of chemical systems, time-resolved CD optical spectroscopies with sub-picosecond to nanosecond resolution have been implemented in various spectral regions^21–26^, in order to investigate the dynamics of peptide chains, metalloproteins and metal complexes^27–30^. Extending these capabilities to core-level spectroscopies allows to identify the role of specific elements within a given enantiomer during a chemical reaction.

Simulations of X-ray CD signals of organic molecules show that the dichroic response of a given element varies with the electronic coupling to substitution groups, its distance from the chiral centre, its local geometry and chemical structure^31–38^. This implies that for identical atoms, e.g. carbon atoms, the CD signal is different for different chemical environments^31–33,36^. This is a great advantages as it adds the dichroic response to the chemical shift allowing to distinguish identical but non-equivalent (due to their different chemical environments) elements. As X-ray absorption edges are often congested, the combination of these two observables can help distinguishing them spectrally, and it is therefore particularly attractive for the study of a large class of organic molecules in solutions^39^. Since these molecules mostly consist of light elements whose core transitions lie between 280 eV (carbon) and 700 eV (fluorine), this calls for ultrashort sources of circularly polarized soft X-ray pulses. Table-top sources based on HHG are starting to be implemented in this photon energy range^11,40^, but the control of their polarization still needs to be demonstrated^41,42^. On the other hand, circularly polarized soft X-ray pulses can routinely be generated at the FERMI FEL in the region of the carbon K-edge^43^.

Most chemical reactions occur in the dark and therefore, the ability to visualize interactions between ground state molecules with element- and enantioselectivity would give a unique degree of insight into their reactivity. Impulsive stimulated Raman scattering (ISRS) is a well-established method to generate coherent vibrations in the ground state of molecules, and in particular, in the low-frequency region where intermolecular motions are often encountered^44^. Here we combine ISRS with ultrafast circularly polarized soft X-ray absorption spectroscopy at the carbon K-edge, to visualize the response of specific carbon atoms in a racemic powder mixture of 4-isobutyl-2-phenylpropionic acid, commonly known as Ibuprofen (IBP), subject to low-frequency coherent vibrations in the ground state. The ISRS triggers the latter, which translate into modulations of the C K-edge absorption. Furthermore, the polarization control of the EUV pulses adds enantiomeric selectivity, tying it to the element-selectivity.

## Results

### Low-frequency Raman modes

**Fig. 1:**
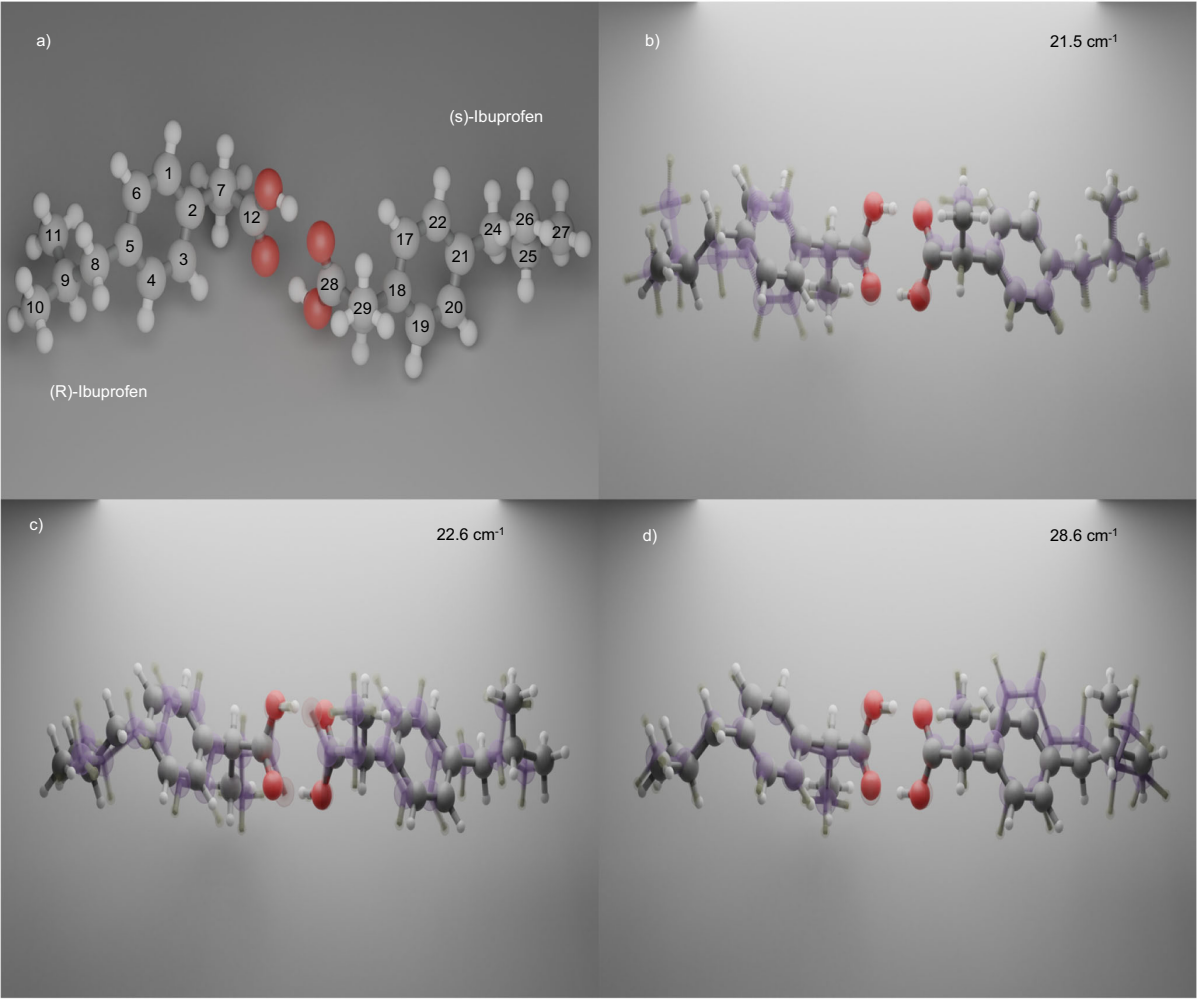
Atom labelling and vibrational modes. **a** The Ibuprofen dimer with the numbering of the carbon atoms. **b**–**d** The three intermolecular modes of the dimer (see Supplementary Movie 1–3). The structures appearing in contrasted and semi-transparent colours, correspond to the minimum and maximum deformations for the calculated intermolecular modes of the dimer.

IBP is an over-the-counter anti-inflammatory non-steroidal drug that is widely used for its analgesic and antipyretic activities^45^. It is chiral and its two enantiomers are labelled (S+) and (R−), with the former being the pharmacologically active one, although the underlying reason is not clear, as for example in the cross-monomer allosteric inhibition, in which (S)-IBP can competitively block the action of one monomer of the cyclo-oxygenase (COX) enzyme, composed by two equal halves^46^. The commercial drug is the racemic mixture of these two enantiomers, whose crystalline phase I ((RS)-IBP) is stable up to the melting point *T*_m_ = 349 K^24^. The two enantiomers form a head-to-tail dimer through intermolecular hydrogen bonds between the carboxyl groups of two adjacent molecules (Fig. 1). The activity of the two enantiomers, and in particular the off-resonance Raman spectrum of IBP has been investigated by several authors^47–50^. The spectrum exhibits several bands in the low-frequency (20–150 cm^−1^) region, but also at high frequencies, in particular in the fingerprint region between 600 and 1700 cm^−1^. The origin of the low-energy vibrations is important for the present study. While previous experimental Raman and theoretical studies mainly focused on the intramolecular vibrations lying above 200 cm^−1 ^^47,51–53^, Hédoux et al. found that the low-energy region^48^ exhibits three spectral features at ~20 cm^−1^, ~50 cm^−1^ and ~80 cm^−1^, which the authors assigned to phonons, i.e. intermolecular collective modes. Lazarevic et al. ^49^ argued that the symmetry properties of the (RS)-IBP crystal structure imply a large number of the intermolecular vibrations, leading them to conclude that the above low-frequency features of the Raman spectrum are spectrally unresolved multi-peak structures.

In the present work, we revisited the low-frequency range of the Raman spectrum of the (RS)-IBP powder (see Supplementary Methods 2 for details) and reproduced the published results, showing a clear feature at ~24 cm^−1^ with an additional weaker peak at ~28 cm^−1^ (see Supplementary Fig. 9). Animations of the IBP low-energy vibrations are reported in Supplementary Movies 1–3. These results were complemented by Fourier Transform Far-Infra-red studies (see Supplementary Methods 2 for details), which exhibit several features up to 70 cm^−1^ and confirms the presence of a weak feature between 20 and 30 cm^−1^ (see Supplementary Fig. 10). More recently, Krausbeck et al. ^54^ simulated the off- (excited at 514.5 nm) and on-resonance (excited at 265.9 nm) Raman spectra of the S-IBP monomer. While the RS dimer shows a low-frequency region that is characterized by several spectral features up to ~150 cm^−1^, the S monomer exhibited only a single feature in the 0 to 200 cm^−1 ^region, around 20–30 cm^−1^. Based on the above-mentioned studies, it is possible to conclude that: (a) resonance enhancement of the Raman spectrum in the lowest frequency region of the S-IBP monomer yields exclusively the modes around 20-30 cm^−1^; (b) in the crystal, these modes contain intramolecular and intermolecular (dimeric) contributions, including phonons. We therefore anticipate that exciting the system with a femtosecond pulse tuned to the region of the first absorption band of IBP would trigger by ISRS, low-frequency modes of both the monomer and the dimer^49,54^.

### Carbon absorption spectrum

**Fig. 2:**
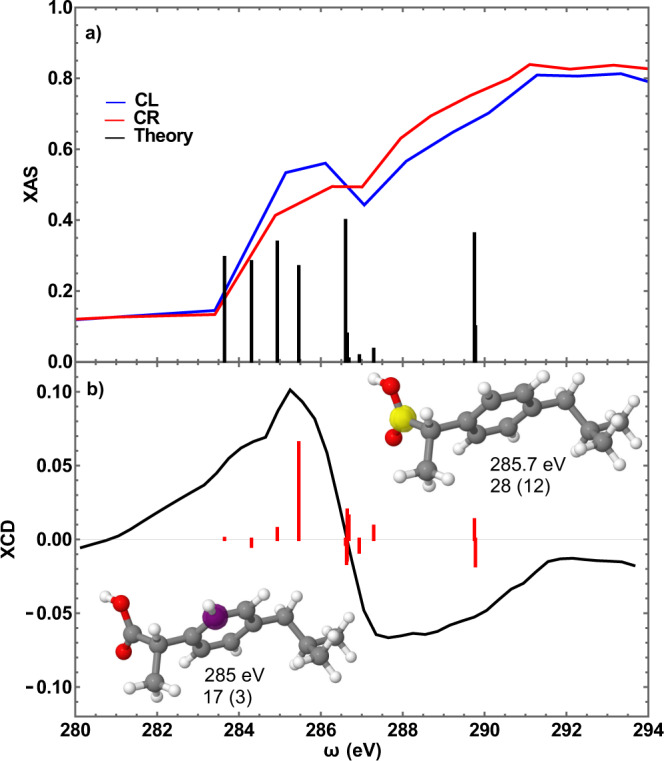
Absorption spectrum and XCD signal. **a** Steady-state carbon K-edge absorption spectrum of S-IBP recorded with circularly left (blue trace) and right (red trace) polarized light. The sticks represent the calculated transition energies and oscillatory strengths for the first core-excited state of each of the 13 carbon atoms in the molecule. Black sticks are the transitions investigated in the present work. **b** Steady-state carbon K-edge X-ray circular dichroism (XCD) signal calculated as the difference of the L and R spectra shown in panel **a**, normalized by their sum. Insets in panel **b** show the selected atoms by the corresponding probe wavelength and their numbering. Numbers in brackets refer to the S enantiomer.

Figure 2a shows the steady-state C K-edge absorption spectra for RC and LC light recorded in a point-by-point scan of the FEL as described in methods section. These spectra exhibit clear differences, namely, the spectrum recorded with LC light shows an enhanced absorption at the edge (285–286 eV) compared to the RC counterpart, while above 287 eV, the spectrum using RC light has a stronger absorption. The difference between these two spectra is shown in Fig. 2b, which represents the C K-edge CD spectrum of the system. It shows the clear dichroic response of the system with a characteristic energy dependence in the region of the C K-edge^32,33,38^, which in principle reflects the response of all C atoms in the system. Further support to this comes from quantum chemical calculations of the C K-edge energies at the cc-pVDZ/RASSCF(6/6) level (details are given in Supplementary Methods 1). Single reference calculations that ignore electronic correlations are poorly suited for core-excited states calculation. CASSCF can achieve a more accurate calculation using multi-determinant wavefunctions optimized within subsets of the molecular orbitals constituting the active space (AS). The calculations were performed for four different AS including different molecular orbitals within the SCF optimization. Increasing the size of the AS was carried out until convergence, chemical plausibility and agreement with experimental spectra were attained (see Supplementary Methods 1). Too small AS’s induce transition energies and moments that strongly depend on the orbital included while the larger ones converge to a more physical result at the expense of a higher computation cost. Supplementary Table 4 gives the K-edge energies of the different C atoms as numbered in Fig. 1a, and Supplementary Fig. 4 shows the shape of the various HOMO’s and LUMO’s. Supplementary Figs. 6 and 7 show their energies and oscillator strengths in the form of stick diagrams for the different AS’s. A rigid shift of 6.4 eV was applied to the core-transition energies in order to match the experimental spectra. AS3 is taken as the most reliable because it shows results comparable to AS2 but is larger than the latter. The atoms that come close to the edge energy around 285 eV are labelled (17,3) and (23,7), (24,8), (25,9), (28,12) and (29,13), which refers to the identical atoms in the two enantiomers. The energy difference between the different C atoms is ascribed to the K-edge chemical shift due to different local environments around the atoms (see Supplementary Methods 1 for details). However, of these only (28,12) near ~285.7 eV and (17,3) near ~285 eV have an appreciable oscillator strength (Supplementary Fig. 7). Figure 2a reproduces the stick diagram due to these atomic transitions. Further to this, we also simulated the CD spectrum (shown in Fig. 2b) following the procedure of ref. ^55^ at the RASSCF(9/8)/cc-pVDZ level. The agreement with the experimental CD spectrum is very good, confirming that inequivalent C atoms exhibit a differential dichroic response at the C K-edge, i.e. the enantiomeric selectivity allows distinguishing different classes of C atoms^33,34^. This is a key point because it implies that in a chemical reaction, e.g. upon binding to a substrate, not all atoms respond in the same fashion, in particular those located on different enantiomers.

### Time-resolved studies

**Fig. 3:**
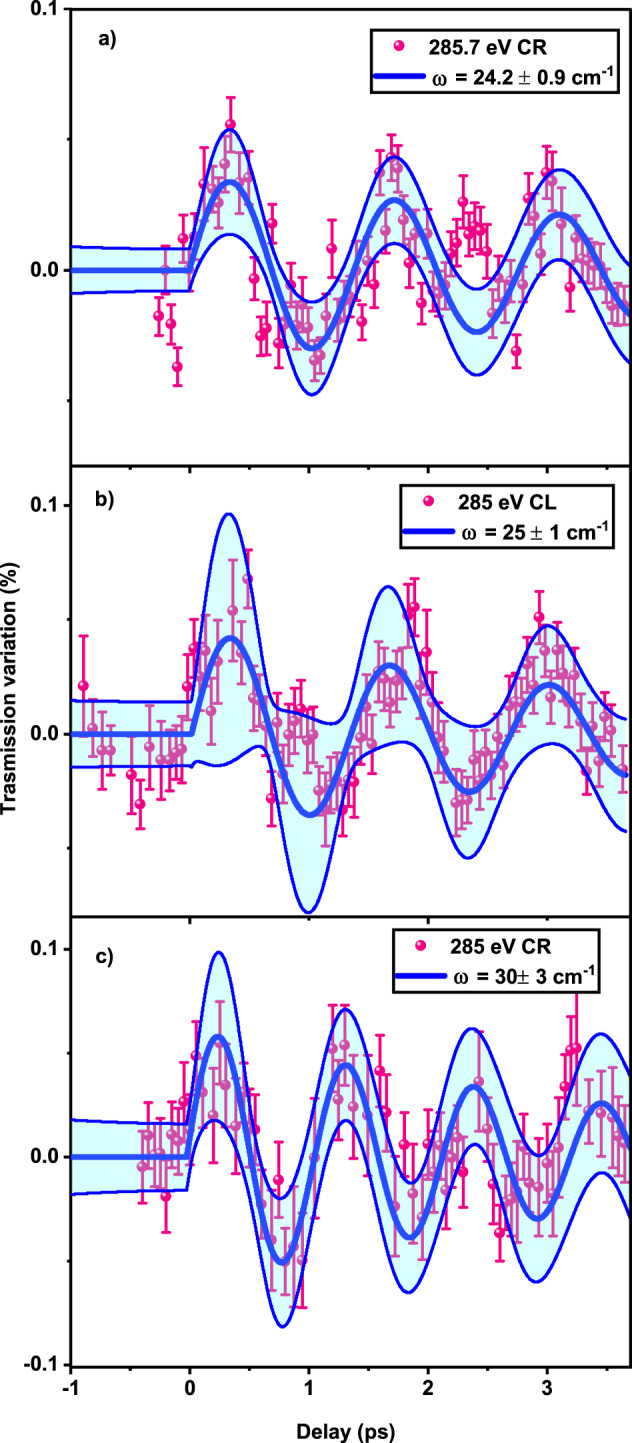
Time-resolved X-ray transmission signal. Time-resolved X-ray transmission signal of the racemic IBP sample measured using probe pulses of: **a** 285.7 eV with circular right polarization: **b** 285 eV with circular left polarization and; **c** 285 eV with circular right polarization. Thick blue lines are fits to the data with damped sinusoidal functions whose parameters are given in Table 1. Shaded cyan areas represents the fit confidence bands. Error bars are calculated as errors of the mean value as described in ref. ^65^.

Figure 3 shows the time evolution of the transmission signal upon impulsive excitation of the system at ~4.7 eV and for different soft X-ray probe photon energies and circular polarizations. All traces reveal periodic intensity modulations (5–10 % amplitude of the signal) around a mean value of the transmission. The data in panel (a) were obtained at a probe energy of 285.7 eV and with RC polarized light. Panels (b) and (c) show time scans acquired using a probe photon energy of 285.0 eV with LC and RC polarization, respectively. All three panels exhibit clear modulations with a sine dependence, as expected for Raman-induced processes^56^. We refrain from plotting the time traces of the CD signal (difference between left and right polarized signal) because, due to the low S/N and the fact that the modes have the same phase, this would completely wash out the modulations. The three traces were fitted to damped sinusoidal functions (described in methods sections), which yield the frequencies and damping constants given in Table 1 and Supplementary Table 1. These frequencies (periods corresponding to ca. 1.1–1.4 ps) are in good agreement with the calculated and measured lowest Raman peaks, also given in Table 1 and Supplementary Table 2. The damping times (1.4–4 ps) of these three modes correspond to 8 to 25 cm^−1^ spectral widths, which explain why they cannot be resolved in the steady-state Raman spectrum (See Supplementary Fig. 9). For technical reasons, the data could not be measured until full damping of the traces, causing important uncertainties on the values of the damping constants.

**Table 1:** Parameters of the fit of the temporal traces using damped sine functions (see Supplementary Table 1 for details)

Probe energy/polarization	Frequency (cm^−1^)	Damping constant (ps)	Calculated (cm^−1^)
285 eV CL	25 ± 1	2.5 ± 1.3	21.5
285 eV CR	30 ± 3	1.5 ± 0.6	28.8
285.7 eV CR	24.2 ± 0.9	3.8 ± 3	22.6

The low-frequency steady-state Raman spectrum exhibits only one band centred around 25 cm^−1^ whose width increase from ~5 cm^−1^ at 100 K to ~10 cm^−1^ at 300 K^49^.

Important to note in Fig. 3 are the following: (a) the difference between the CR and CL signal at 285 eV clearly points to an enantioselectivity of the molecular vibrations by the polarized X-ray pulse, via the element-selectivity; (b) the difference between the 285 eV and 285.7 eV CR traces, shows that by tuning even slightly (0.7 eV) the probe energy, the response of the system significantly changes. These observations highlight the capability of our experimental approach to distinguish between identical elements having different chemical environments. It also shows that these elements respond differently to the low-frequency deformations of the molecule.

## Discussion

It is interesting to compare the present approach to other methods that can be chromophore-sensitive, in particular vibrational optical activity (VOA). VOA (infra-red CD) has been shown capable of determining absolute configuration of small organic chiral molecules and the supramolecular chirality of biomolecules such as the protein secondary structure and the DNA helicity^56–58^. However, VOA spectroscopy suffers from the intrinsically weak (electric-dipole forbidden) chiral signal and is affected by the intense achiral (electric-dipole allowed) background. These hinder its transfer to dynamical (time-resolved) studies. In order to enhance chiral-to-achiral contrast ratio, non-linear methods, such as chiral vibrational sum-frequency generation (SFG)^57–59^ and CARS (coherent anti-Stokes Raman scattering)-ROA^60^ spectroscopies, have been implemented and allow chiroptical time-resolved studies in isotropic media. However, none of these studies has reached the low-frequency range of intermolecular modes, and they additionally lack element-specificity.

The present results suggest a strategy to identify which atoms are most affected by molecular deformations (in the present case, low-frequency vibrational modes) in a given enantiomer subsequent to a perturbation (photon, chemical reaction, etc.) that induces a deformation. This calls for more systematic investigations. It also paves the way for the direct investigation of ground state molecular reactions via the intermolecular vibrational dynamics, with the potential to understand the marked differences in chemical reactivities of enantiomers. For example, it is known that the S-form of IBP preferentially binds to cyclodextrins, with a direct effect on its vibrational modes. More generally, such a detailed level of understanding may lead to new design strategies of (bio)active molecules such as vibrational/deformational engineering by the use of isotopes to modify the vibrational behaviour of an identified atom or molecular moiety but keeping unaltered the electronic properties. Finally, the actinic pulse that induced the ISRS process could trigger photoactive reactions^27,30^ and the present super-selective pump-probe approach could track the behaviour of selected atoms during the photochemical process.

## Methods

### Experimental set-up and procedures

**Fig. 4:**
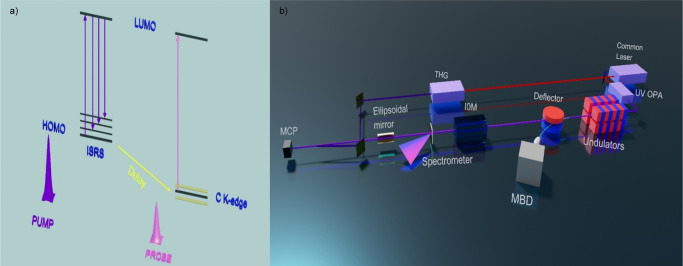
Sketch of the ISRS process and of the FEL layout. **a** Pulse scheme of the experiment with the relevant energy levels: The 4.7 eV pump pulse (purple pulse) generates a coherent superposition of vibrational levels in the HOMO by impulsive stimulated Raman scattering (ISRS). These modes are then probed with element-selectivity by the soft X-ray pulse (light purple pulse) tuned to the carbon K-edge. **b** The experimental layout. A 786 nm femtosecond laser pulse is split and delivered to an optical parametric amplifier (OPA) where it is up converted to generate the ~4.76 eV pulse that initiate the Free Electron Laser process, as well as a frequency-tripling (THG) to generate the 4.7 eV pump pulse. The seeded electron bunch is propagated through a chain of apple II undulators, allowing control of the polarization and energy of the soft X-ray photons. An ionization chamber (IOM) and a spectrometer are used to record on a pulse-to-pulse base the characteristics of FEL beam. An ellipsoidal mirror is ultimately used to focus the FEL beam on the sample.

The UV–Vis absorption spectrum of IBP in solution, recorded at room temperature, is shown in Supplementary Fig. 3. Its first absorption band lies between 250 and 280 nm, with structured features related to the different electronic transitions of the aromatic ring. The spectrum is well reproduced by theoretical calculations shown as a stick spectrum (details in Supplementary Methods 1). The experimental scheme used in the present study is shown in Fig. 4a. The pump-probe experiment was conducted at the EIS-TIMEX^61^ end-station of the FERMI FEL^62^ (Trieste, Italy). The sample was photoexcited with laser pulses at a photon energy of 4.7 eV, resonant with the IBP absorption (Supplementary Fig. 3), an energy/pulse below 1 μJ and pulse duration of 80 fs. The pump beam is focused onto a spot size of 90 × 90 μm^2^ full-width-at-half-maximum (FWHM). It impinges the sample at an angle of 10 degrees, relative to the surface normal, while the FEL probe pulse is at normal incidence. This pump pulse generates low-frequency modes of the IBP dimer by impulsive stimulated Raman scattering (ISRS)^44^. Given the spectral and temporal width of the UV pulse inducing the ISRS process, all modes below 370 cm^−1^ could be coherently excited, including those around 20–30 cm^−1^ that dominate the low-frequency resonance Raman spectrum^54^. Probing molecular motion is possible only if the molecular vibrations affect the energy (chemical shift) and/or intensity the K-edge absorption spectra of the different C atoms. The soft X-ray FEL photon energies were chosen to match the transitions identified by the theoretical calculations in the C K-edge region (see Supplementary Methods 1 and black sticks in Fig. 2a). To prevent long-term IBP photo degradation and optimize the spatial overlap, the FEL spot size was set to 80 μm FWHM and the energy/pulse was reduced to about 160 nJ. The estimated FEL pulse duration is about 30 fs FWHM. Sample degradation was excluded by visual inspection performed through a Questar QM100 tele-microscope^61^. In order to minimize radiation damage effects (detectable as a monotonic increase in the measured transmission of the spot), after each time scan the sample was moved to a pristine region.

The soft X-ray intensity transmitted through the sample was detected by a Multi-Channel Plate (MCP) placed on the FEL beam axis and calibrated using an identically hydrophilized silicon nitride membrane. The calibration was performed by recording the signal level (I_1_) on the MCP as a function of the incoming FEL intensity (*I*_0_) and modelling the trend by a second order polynomial. This way, it is possible to estimate the expected *I*_1_ for any given *I*_0_ value and to calculate the IBP layer transmission shot-by-shot, as the ratio between the measured I_1_ and the one calculated with the second order polynomial defined above. The procedure was repeated for every probe energy/polarization (experimental configuration).

Time traces were collected by continuously scanning the pump-probe delay (at a velocity of 0.5 ps/s) and recording the transmission of the soft X-ray beam. The measurements were repeated three times for each experimental configuration. The data were subsequently merged and binned in 50 fs steps; the data points displayed in the time traces are the mean values of the bin content while error bars are the standard deviations of the bin entries.

Supplementary Fig. 2 shows the pump laser-off shot-by-shot transmission of IBP acquired over 3500 FEL shots. These scans correspond to a total acquisition time of 350 s each. They show the long-term stability of the system and data acquisition. They do not exhibit any features, as expected from removing the pump pulse. These scans need to be compared to the acquisition time of a pump-probe temporal traces of about 30 s.

The steady-state carbon K-edge absorption spectra (Fig. 2a) were obtained acquiring and averaging 3500 shots for each FEL photon energy and polarization. FEL photon energies between 293.5 and 285 eV were generated by high gain harmonic generation (HGHG) of the 60th harmonic of the seed laser was tuned between 4.9 and 4.7 eV in steps of about 0.02 eV. Energies range between 283.7 and 279.2 eV were instead obtained by amplifying the 55th harmonic of the seed laser tuned between 5.16 and 5.1 eV in steps of about 0.02 eV. In both configurations, the polarization was varied by changing the phase of the magnetic field inside the apple II undulators. The X-ray Circular Dichroism spectrum shown in Fig. 2b of the text is the difference between the spectra obtained in the two circular polarizations normalized by their sum.

### Sample preparation

The sample was prepared by dissolving 3 mg of an IBP racemic mixture into 1 mL of ethanol (Sigma-Aldrich, >99.8%). The solution (1 µL) was deposited on a commercial Silicon Nitride membrane (Silson, 25 mm^2^ surface area, 100 nm thickness). In order to obtain the total coverage of the silicon nitride membrane. Reactive Ion Etching (RIE) plasma oxygen process was used in order to modify surface wettability (radio frequency power 20 W, O_2_ flow 30 sccm, exposure time of 5 minutes). Water contact angle measurements showed that this treatment reduced the water contact angle from 85.1° to 28.5°, as shown in Supplementary Fig. 1. After the solvent evaporation, IBP is expected to recrystallize to its native form. Due to the steric encumbrance of the most probable crystalline form at room temperature^63^, we expect to have the phenyl rings almost parallel to the sample surface, with no or only a weak dependence on the employed deposition process^64^. Under these conditions, considering the density to be 1.1 g/cm^3^^64^, we estimate an IBP layer thickness of about 100 nm.

### Fit of the time traces

The data points of the signal as a function of time were fit using the function:

1 $$y\left(t\right)={y}_{0}+\theta \left(t\right)A{e}^{-\frac{t}{\tau }}{{\sin }}\left(\frac{2\pi t}{T}\right)+{mt}$$

Where *θ*(*t*) is the Heaviside theta function. *A* is amplitude of the variation, *τ* is the decay time, and *T* is the oscillation period. Table 1 shows the fitted values for the three data series. Data shown in the text have been obtained by subtracting the small, linearly varying background and normalizing the values for the unperturbed transmission. The confidence bands of the fit have been calculated using the propagation of uncertainty on the fitted quantities according to the following formula.

2 $$u\left(y,t\right)=	 \surd \left({\left(\frac{\partial y}{\partial {y}_{0}}\right)}^{2}{u}^{2}\left({y}_{0}\right)+{\left(\frac{\partial y}{\partial m}\right)}^{2}{u}^{2}\left(m\right)+{\left(\frac{\partial y}{\partial A}\right)}^{2}{u}^{2}\left(A\right)\right.\\ 	 \left.+{\left(\frac{\partial y}{\partial \tau }\right)}^{2}{u}^{2}\left(\tau \right)+{\left(\frac{\partial y}{\partial \nu }\right)}^{2}{u}^{2}\left(\nu \right)\right)$$

where *u* are the uncertainties of the fitted quantities, given in Table S1, and $\nu=\frac{2\pi }{T}$. The bands in Fig. 3 corresponds to ±2*u*(*y*,*t*), where the factor 2 assumes a 95% coverage probability.

## Supplementary information

Supplementary Information

Description of Additional Supplementary Files

Supplementary Movie 1

Supplementary Movie 2

Supplementary Movie 3

## Data Availability

The shown data are available under restricted access for weight reasons, access can be obtained by contacting the authors.
