# Supplementary material for: Element- and enantiomer-selective visualization of molecular motion in real-time
Source: Nat Commun. 2023 Jan 24;14:386. doi: 10.1038/s41467-023-36047-5 (PMC9873934; doi:10.1038/s41467-023-36047-5)
Supplement: Supplementary file 1 — Supplementary Information [file 41467_2023_36047_MOESM1_ESM.pdf]

Element- and enantiomer-selective visualization of molecular motion in real-time

R. Mincigrucci[1,*,⸸], J. R. Rouxel[2,3#,⸸], B. Rossi[1,4], E. Principi[1], C. Bottari[1,5], S. Catalini[6,7,8], J. S. Pelli-Cresi[1], D. Fainozzi[1,5], L. Foglia[1], A. Simoncig[1], A. Matruglio[9], G. Kurdi[1], F. Capotondi[1], E. Pedersoli[1], A. Perucchi[1], F. Piccirilli[1], A. Gessini[1], M. Giarola[10], G. Mariotto[11], M. Oppermann[12], S. Mukamel[13], F. Bencivenga[1], M. Chergui[2,^], C. Masciovecchio[1,+]

⸸ Authors contributed equally

1 Elettra Sincrotrone Trieste S.C.p.A., Strada Statale 14 - km 163,5 in AREA Science Park 34149 Basovizza, Trieste ITALY
2 Lausanne Centre for Ultrafast Spectroscopy (LACUS), École Polytechnique Fédérale de Lausanne, CH-1015 Lausanne, Switzerland
3 Univ de Lyon, UJM-Saint-Etienne, CNRS, IOGS, Laboratoire Hubert Curien UMR 5516, Saint-Etienne F-42023, France
4 Department of Physics, University of Trento, Via Sommarive 14, 38123, Povo, Trento
5 Department of Physics, University of Trieste, Trieste, Italy
6 European Laboratory for Non-Linear Spectroscopy (LENS), Università di Firenze, 50121 Florence, Italy
7 Department of Physics and Geology, University of Perugia, 06123 Perugia, Italy
8 CNR-INO, Consiglio Nazionale Delle Ricerche, Istituto Nazionale di Ottica, Largo Fermi 6, 50125, Florence, Italy
9 CERIC-ERIC Strada Statale 14 - km 163,5 in AREA Science Park 34149 Basovizza, Trieste ITALY
10 Centro Piattaforme Tecnologiche, University of Verona, Policlinico GB Rossi, Ple. L.A. Scuro, 10, 37134 Verona (Italy)
11 Department of Computer Science, University of Verona, Strada le Grazie 15, 37134 Verona (Italy)
12 Department of Chemistry, University of Basel, Klingelbergstrasse 80, 4056 Basel, Switzerland
13 Department of Chemistry and physics and astronomy, University of California Irvine, Irvine, California 92697, United States

* riccardo.mincigrucci@elettra.eu
# jeremy.rouxel@univ-st-etienne.fr
^ majed.chergui@epfl.ch
+ claudio.masciovecchio@elettra.eu

Supplementary Figures

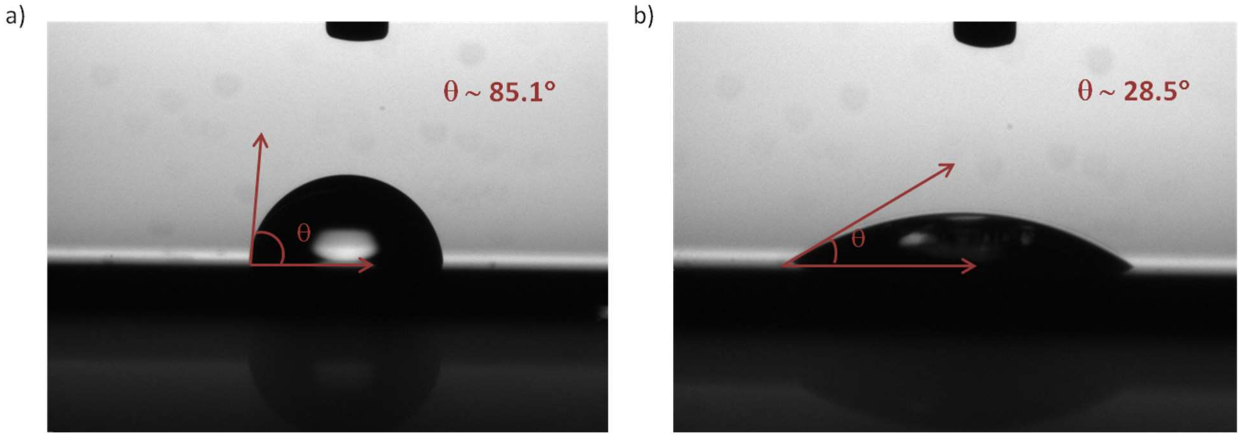

Supplementary Figure 1- Hydrophilization. Water contact angle measurement carried out on the Si_3N_4 membrane: a) before and b) after the hydrophilization process.

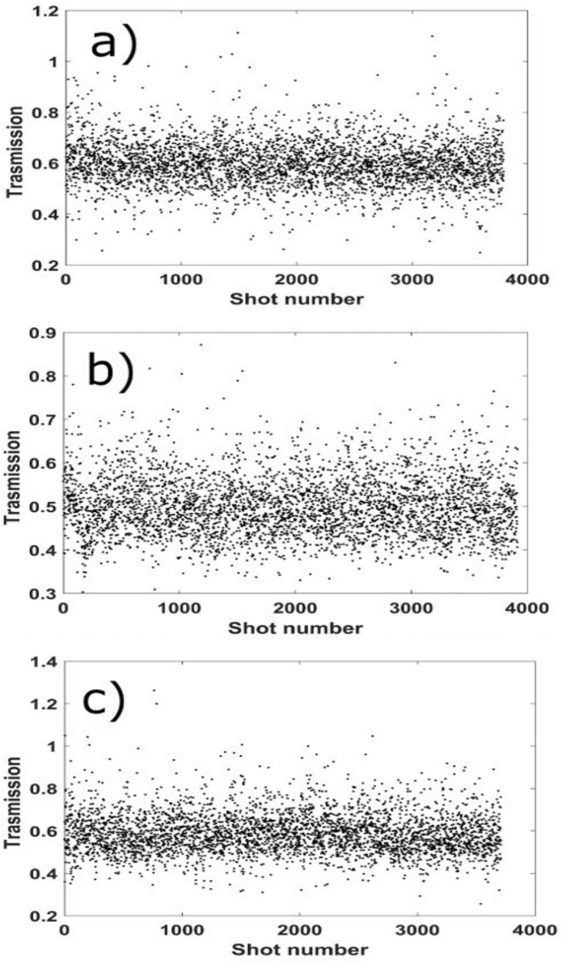

Supplementary Figure 2 – Static sample trasmission. Sample transmission without pump in the three employed configurations: 285.7 eV CR polarization (panel a), 285 CL polarization (panel b) and 285 eV CR polarization (panel c).

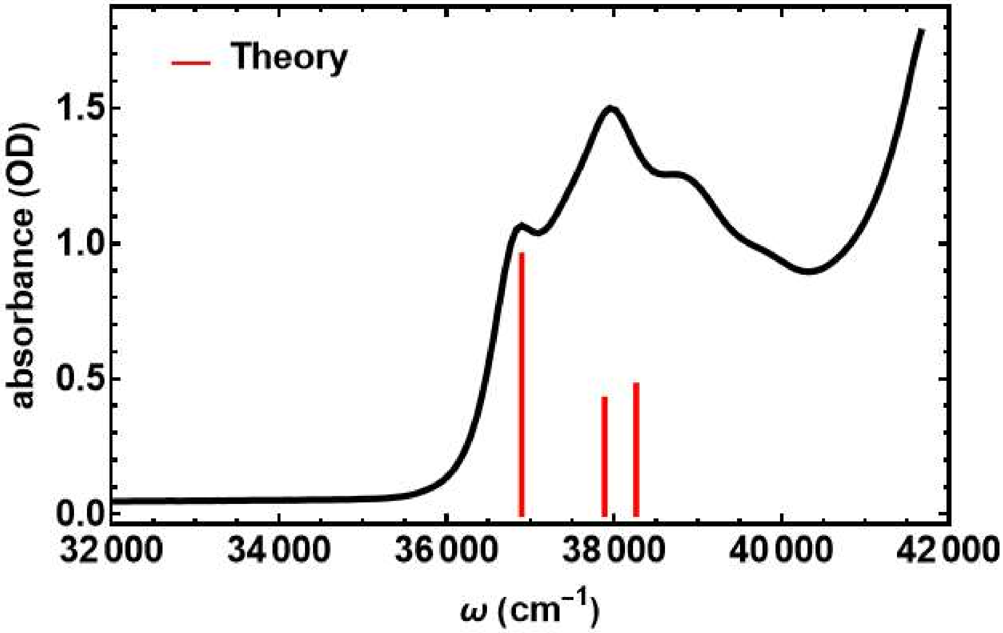

Supplementary Figure 3 - Ultraviolet absorption spectrum. Ultraviolet absorption spectrum of the ibuprofen in an ethanol solution at room temperature. The red trace is the calculated spectrum shifted by 3.5 eV to match the experimental one.

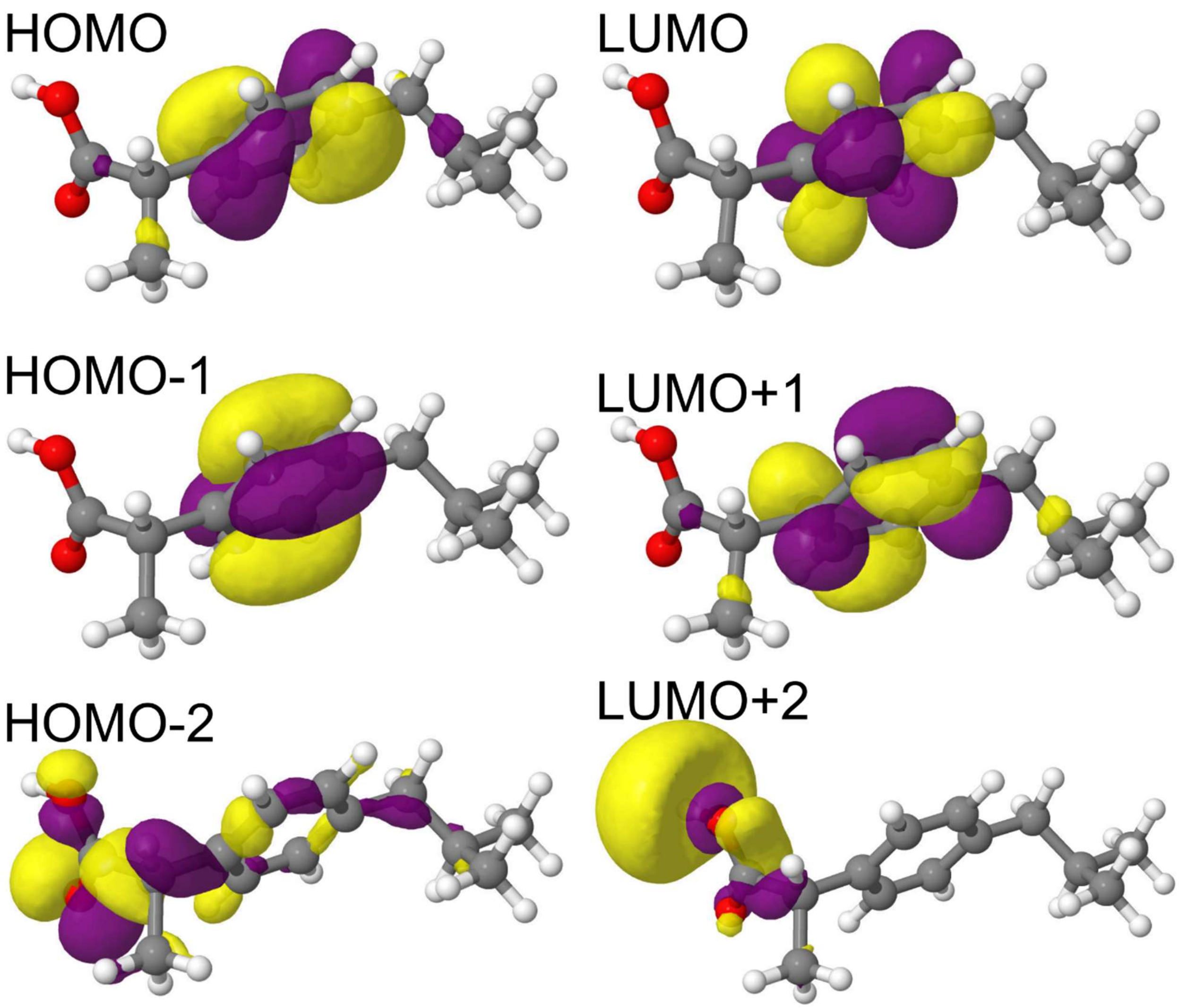

Supplementary Figure 4 – IBP orbitals. Orbitals of the IBP involved in the transitions shown in Supplementary Figure 3. The displayed isovalues are ±0.03, with purple and yellow being the negative and positive isosurfaces, respectively.

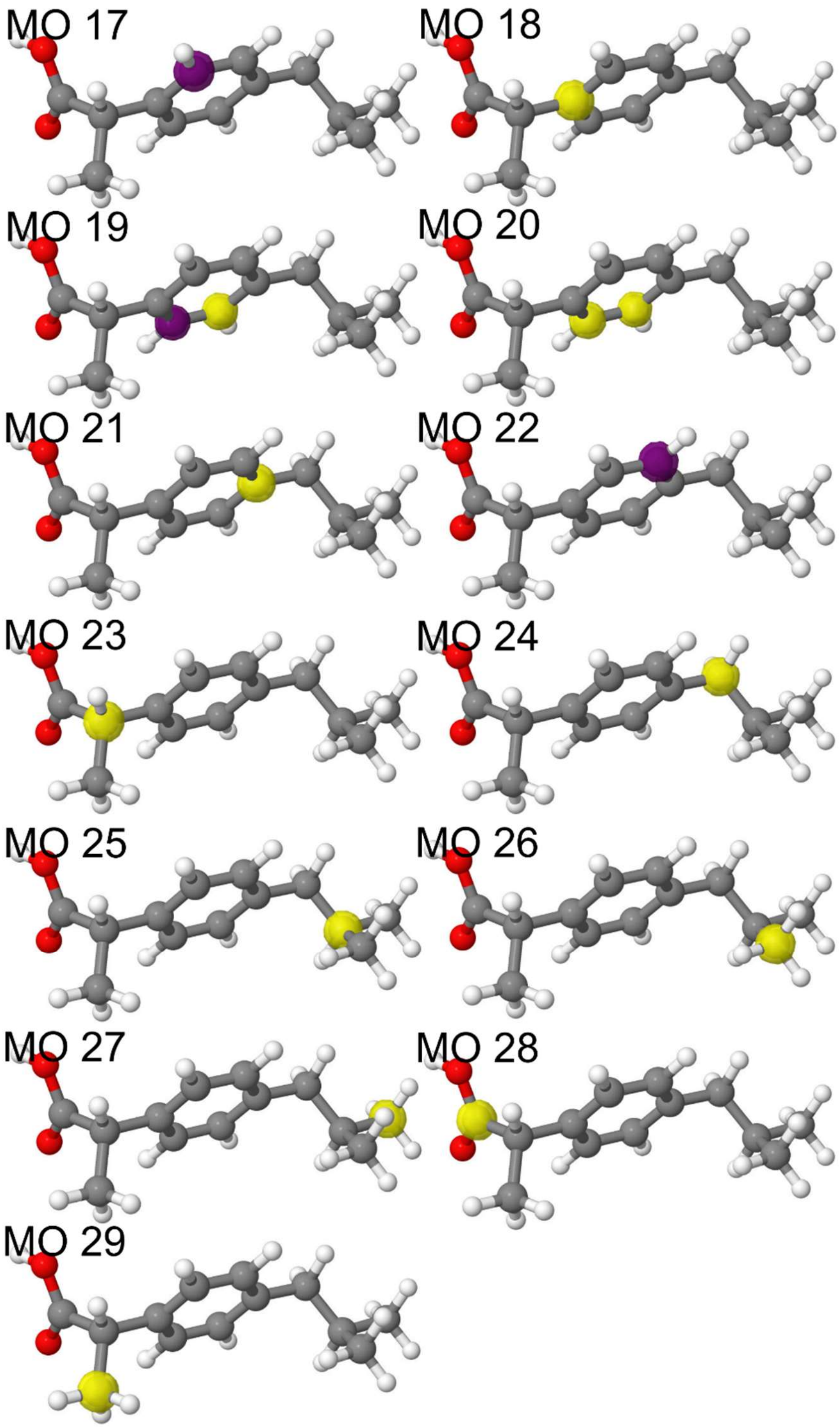

Supplementary Figure 5 – Core Molecular Orbitals of the IBP (isovalue ±0.03). The core MOs are labeled by the number of the carbon atoms in Fig. 1 of the main text. The chosen label corresponds to the atom having the highest electronic density for that orbital, but the core-orbitals also have some small delocalization to nearby atoms. This is especially visible for MO 19 and 20 that are delocalized over atoms 19 and 20. The core-excited states are calculated by rotating one by one these orbitals and restricting their occupation to single occupancy in the RASSCF calculation.

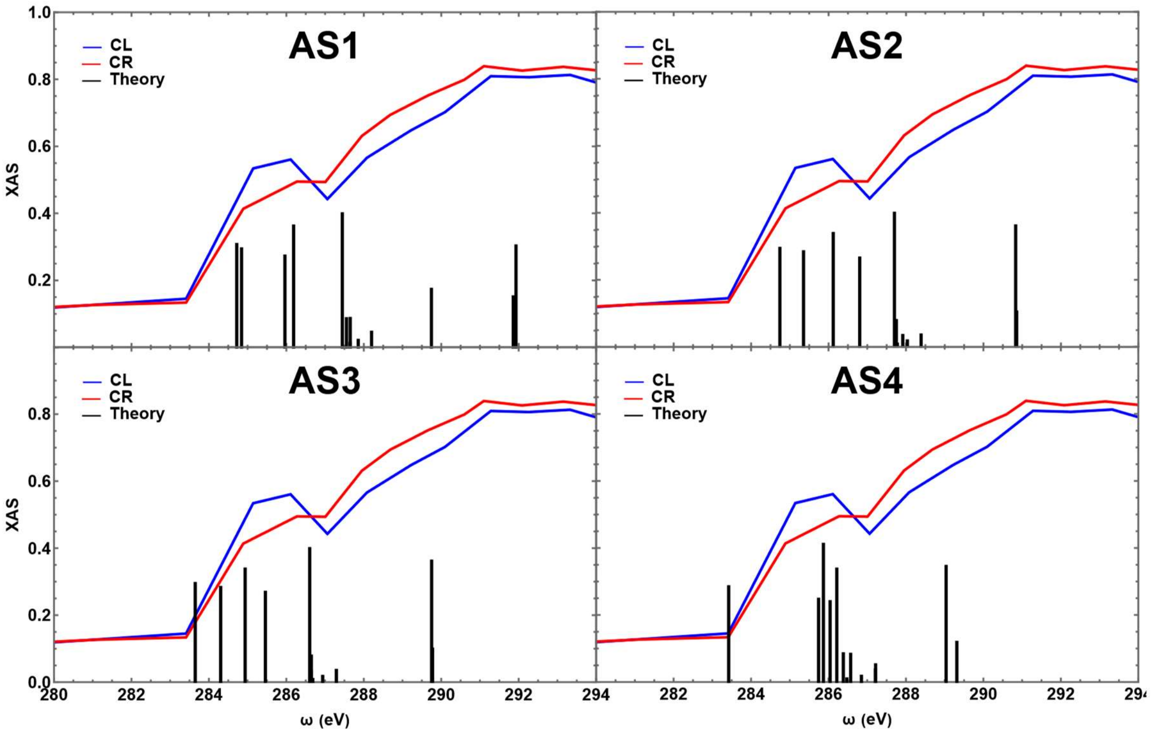

Supplementary Figure 6 – Core absorption spectra as a function of active spaces. The experimental C K-edge spectra for the two circular polarizations recorded point-by-point. Sticks represent the calculated C K-edge absorption transitions obtained for different active spaces.

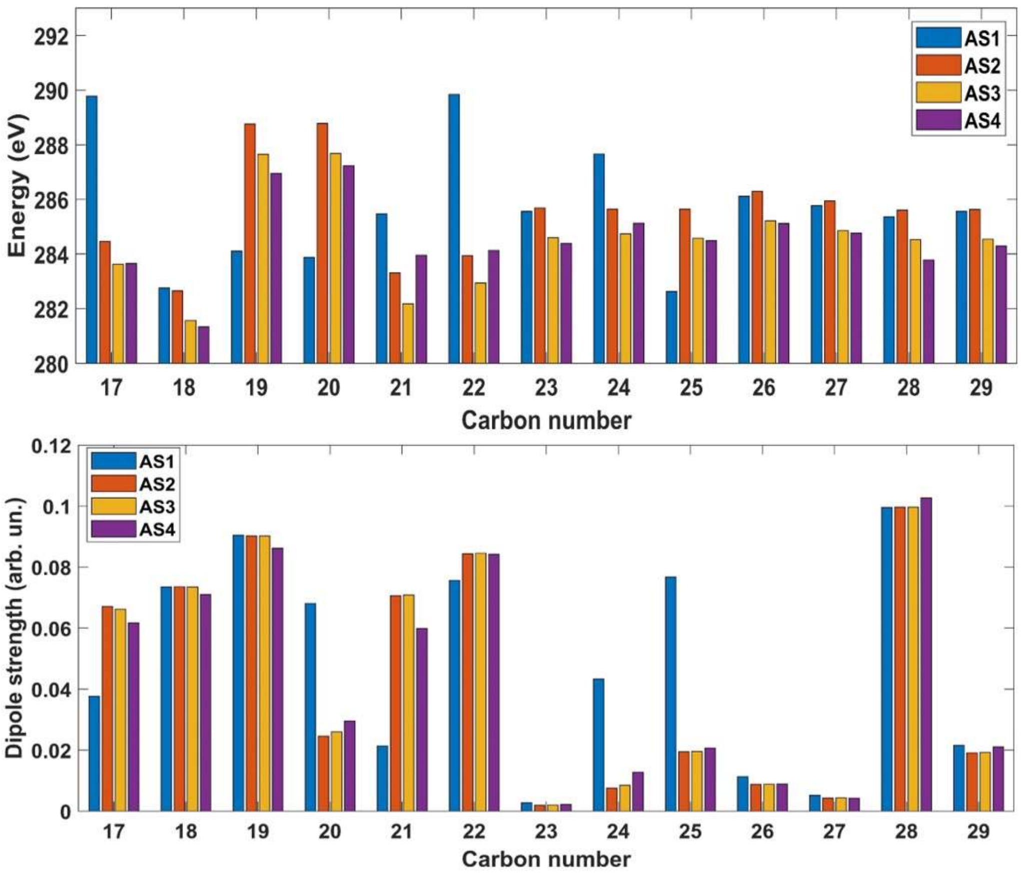

Supplementary Figure 7 – Core transition energies and dipole strengths. Top: Comparison of the core excited states energies for the different active space calculations. Bottom: Comparison of the dipole strengths.

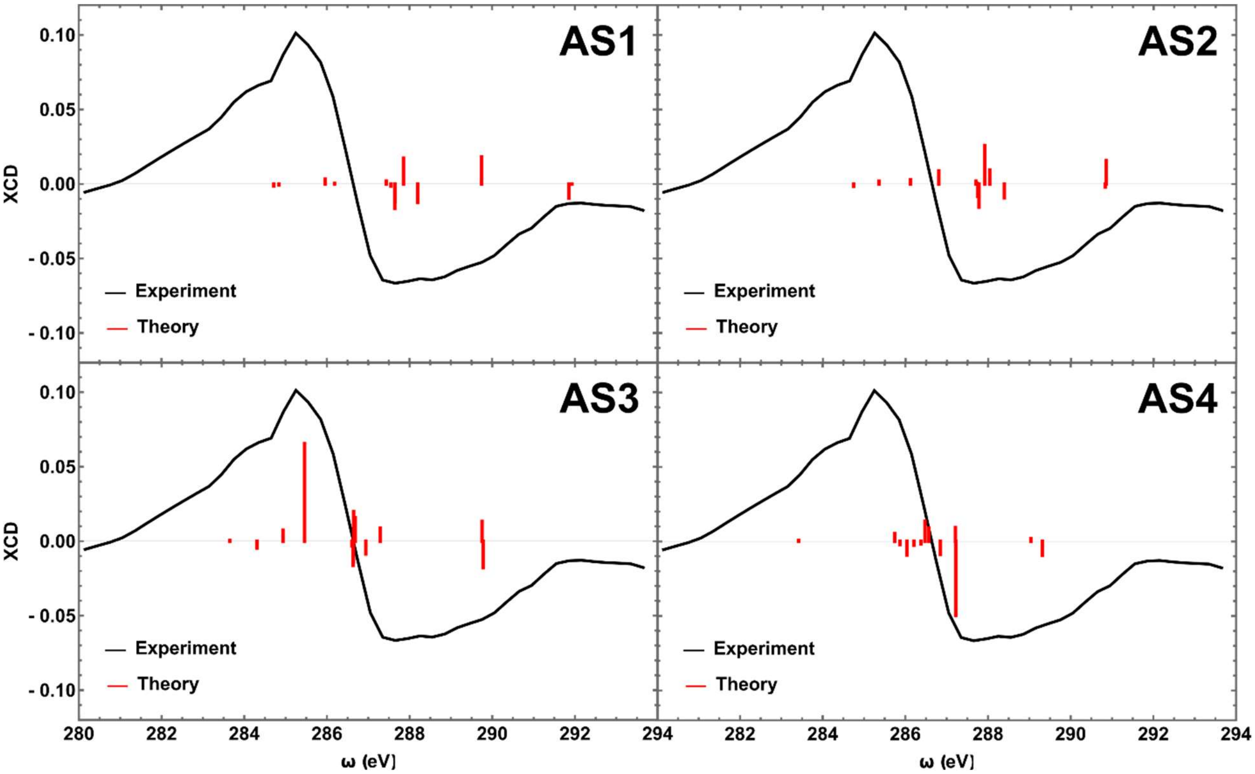

Supplementary Figure 8 –Stick CD spectra obtained for different choices of active space.

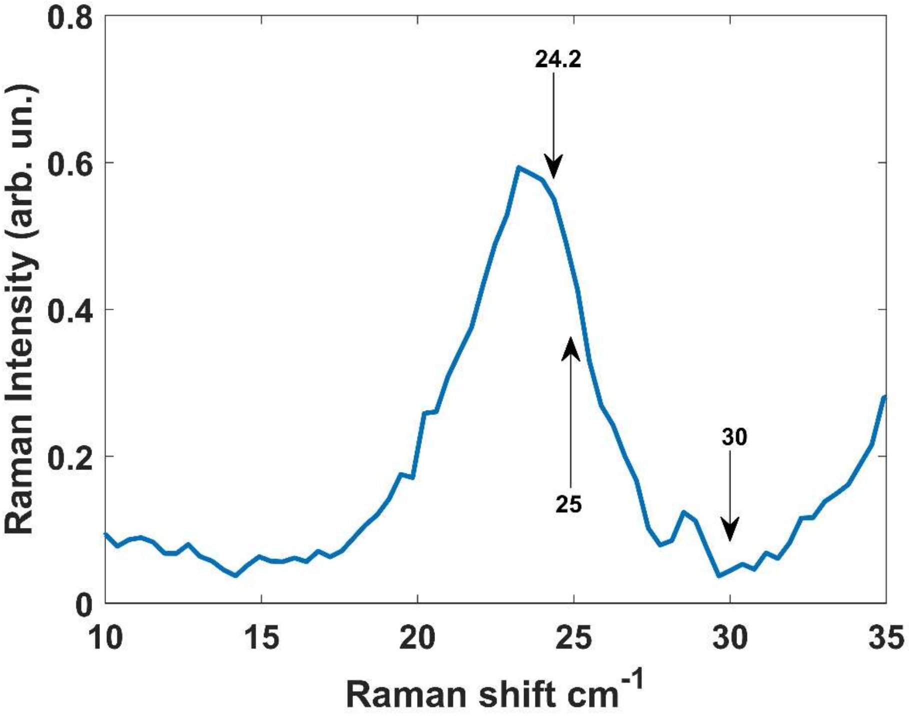

Supplementary Figure 9 – Raman spectrum. Measured Raman spectrum (solid blue line). The black arrows indicate the frequency modes obtained in our experiment.

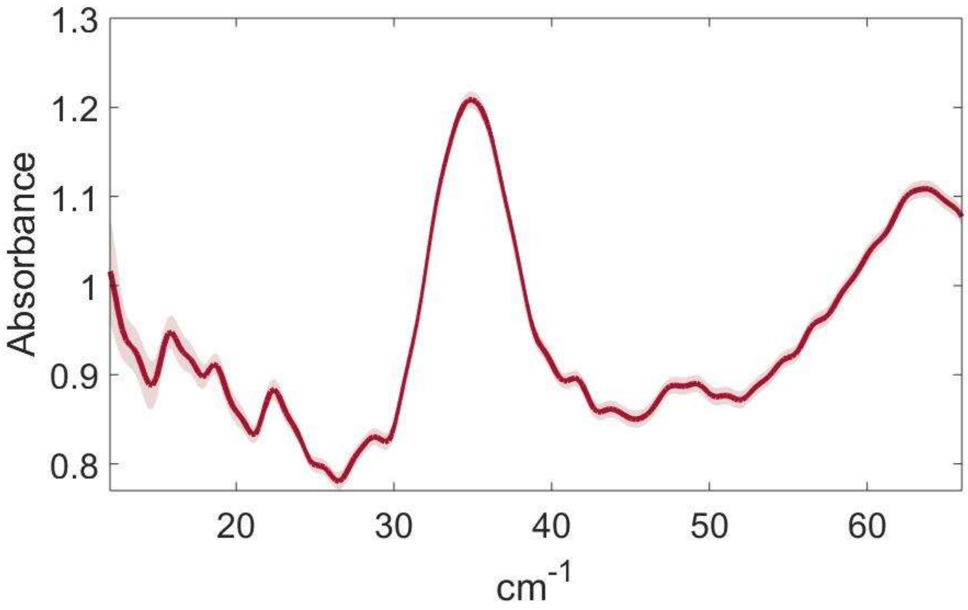

Supplementary Figure 10- Terahertz (THz) spectrum. THz absorption spectrum (red solid line) of a 500 µm-thick IBP powder. The shaded area represents the error in the spectrum determination.

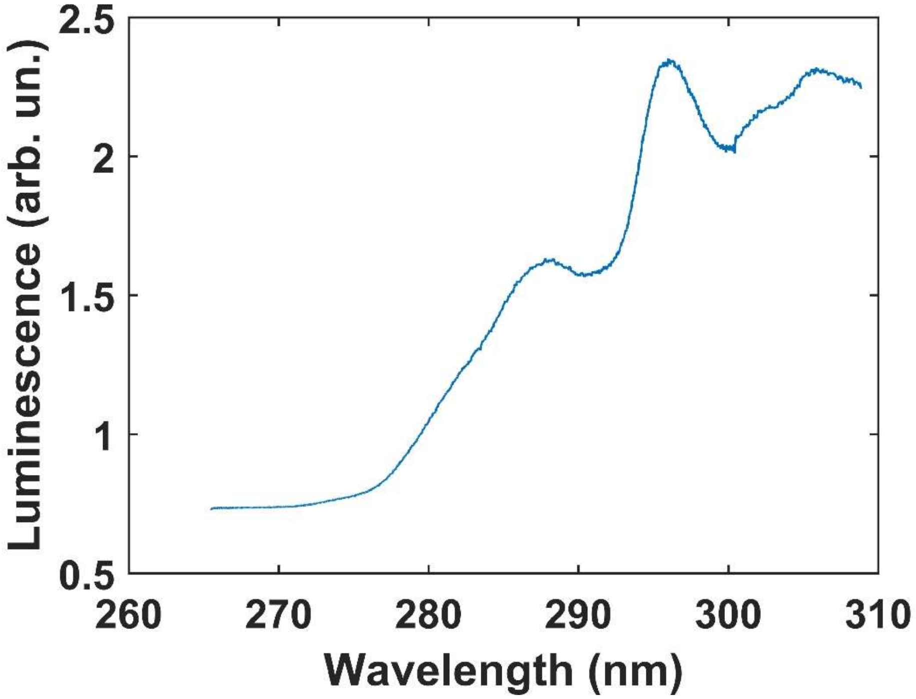

Supplementary Figure 11 – Photoluminescence spectrum. Photoluminescence spectrum (solid blue line) of an IBP powder obtained exciting the sample with synchrotron radiation peaked at 262 nm.

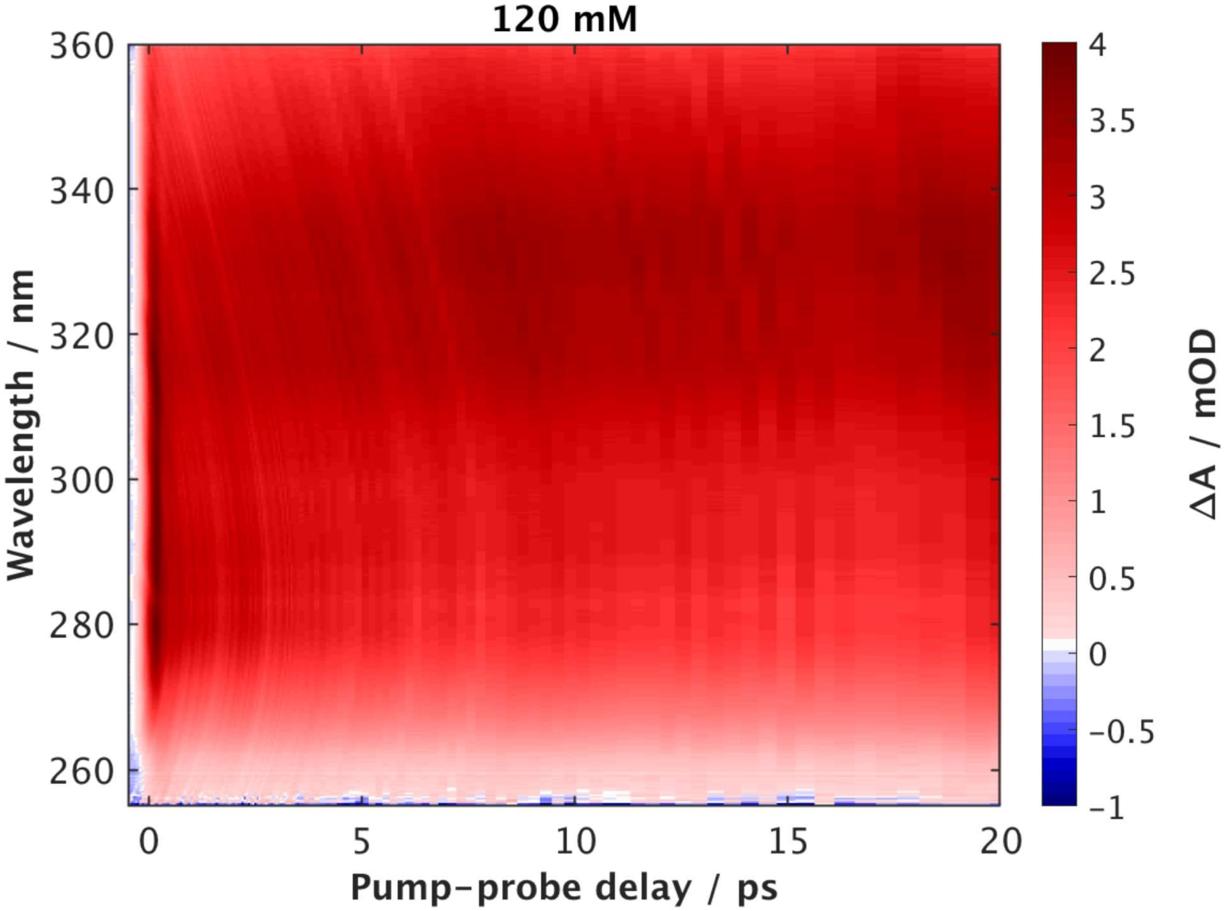

Supplementary Figure 12 - Time-wavelength plot of 120 mMol IBP in solution excited at 266 nm.

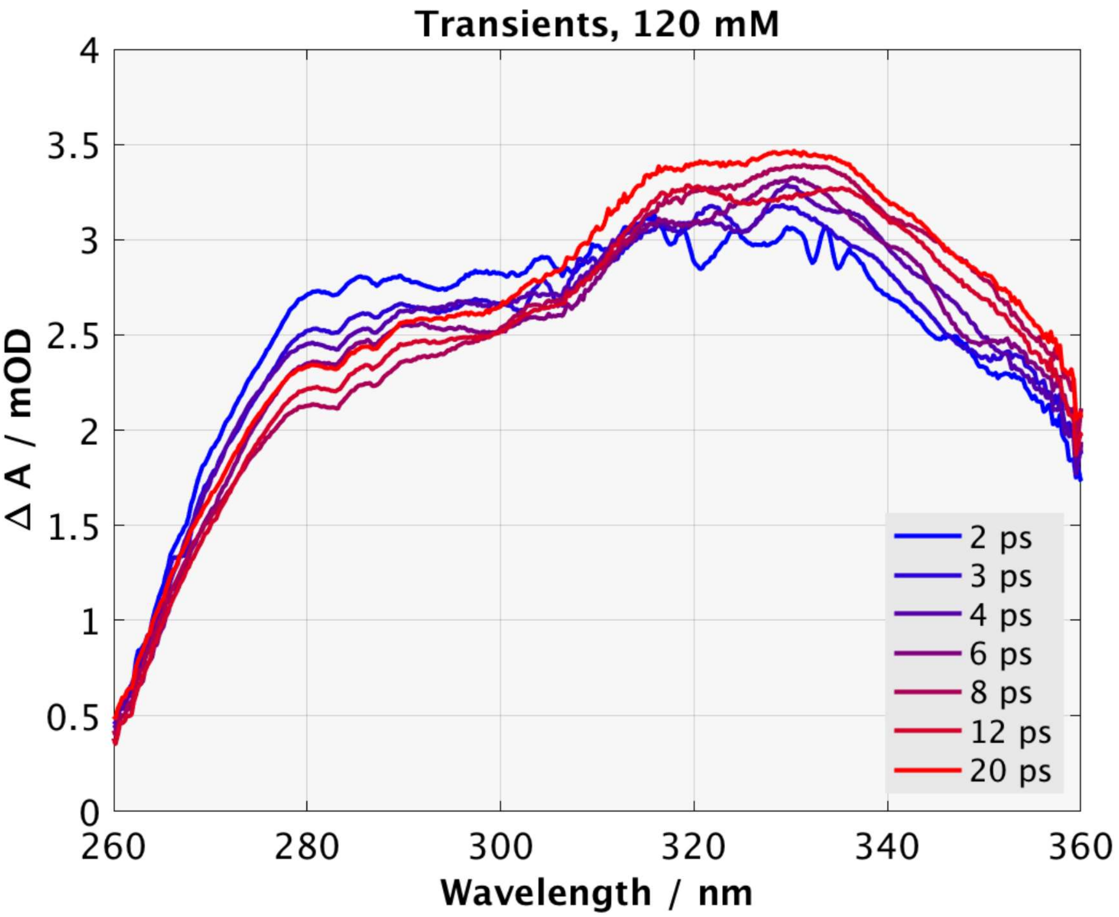

Supplementary Figure 13 - Transient absorption spectra of 120 mMol IBP in solution excited at 266 nm.

Supplementary Tables

Probe	y_0(%)	A (%)	τ (ps)	ν (cm^{-1})	m (%)
285.7 eV CR	0.506 ± 0.004	0.02 ± 0.01	3.8 ± 3	24.2 ± 0.9	0.002 ± 0.002
285 eV CL	0.689 ± 0.007	0.02 ± 0.03	2.5 ± 1.3	25 ± 1	0.001 ± 0.002
285 eV CR	0.560 ± 0.008	0.03 ± 0.02	1.5 ± 0.6	30 ± 3	0.005 ± 0.004

Supplementary Table 1 – Fit coefficient. The table reports the best fit values for the parameters in the function $y(t)=y0+ \theta(t)*A*\exp(-t/ \tau) *\sin(2*\pi/T*t)+m*t$ used to fit the experimental traces. y0 is the unperturbed value for the transmittivity of the IBP layer, θ(t) is the Heaviside theta function, A is the amplitude of the oscillation induced by the pump pulse, τ is the decay time of the oscillatory mode, T is the period of the oscillation converted in cm-1 in the present table and m is the coefficient for the linearly varying background. All fit parameters were free.

Mode number	*Raman Shift (cm^{-1})*	*Mode number*	*Raman Shift (cm^{-1})*
1	7.5	16	120.1
2	10.1	17	138.2
3	21.5	18	155.3 (162.2 [b])
4	22.6	19	171.7
5	28.8 (24.8 [a])	20	183.5
6	41.8 (43.9 [b] / 41.7 [a])	21	210.8
7	49.0 45.6 [a])	22	216.2
8	50.5 (50.3 [b])	23	223.0
9	51.9 (52.3 [a])	24	224.0
10	53.1 (58.9 [b] / 58.9 [a])	25	232.7
11	68.5 (65.6 [b])	26	234.6
12	70.3 (69.9 [a])	27	237.7
13	97.7 (83.5 [b])	28	246.7
14	107.0 (107.7 [b])	29	253.7
15	115.4	30	260.3

[a] Raman scattering spectra of (RS)-Ibuprofen measured at 100 K from ref. [2]
[b] Calculated frequencies for the S-isomer from ref. [2]

Supplementary Table 2 – Vibrational modes. First 30 modes calculated on the Ibuprofen dimer. All the modes listed here exhibit an intermolecular character. Number in brackets represent experimental and calculated Raman frequencies from ref. [2]**.**

	273 nm transition	266 nm transition	263 nm transition
Configuration			
222000	0,00	0,00	0,00
22b0a0	**0,25**	0,00	0,00
22a0b0	**0,25**	0,00	0,00
2b2a00	**0,24**	0,00	0,00
2a2b00	**0,24**	0,00	0,00
b220a0	0,00	0,00	**0,19**
a220b0	0,00	0,00	**0,19**
22ab00	0,00	**0,17**	0,01
22ba00	0,00	**0,17**	0,01
2a20b0	0,00	**0,12**	0,01
2b20a0	0,00	**0,12**	0,01
220200	0,00	**0,12**	0,00
2ab020	0,00	0,00	**0,12**
2ba020	0,00	0,00	**0,12**
202ba0	0,00	0,00	**0,10**

202ab0	0,00	0,00	**0,10**
b22a00	0,00	**0,09**	0,00
a22b00	0,00	**0,09**	0,00
2a200b	0,00	0,00	**0,08**
2b200a	0,00	0,00	**0,08**
22b00a	0,00	**0,05**	0,00
22a00b	0,00	**0,05**	0,00

Supplementary Table 3 – Electronic configurations. Main configurations contributing to the transitions centred at 273 nm (4.542 eV), 266 nm (4.66 eV) and 263 nm (4.733 eV) from the cc-pVDZ/RASSCF(6/6) computation. The first column shows the configurations: the six characters represent the HOMO-2, HOMO-1, HOMO, LUMO, LUMO+1 and LUMO+2 orbitals. The value 2 means a full occupancy of the orbital, while 0 signify that no electrons are in the orbital. Instead, a and b refer to single occupancy with spin up (a) and down (b). The remaining columns indicate the expansion coefficient [28] of the configurations for each of the three valence excited states**.**

	AS1	AS2	AS3	AS4
17	284.718	287.75	286.644	286.571
18	284.843	284.742	283.648	283.422
19	289.745	287.916	286.641	287.216
20	291.929	286.12	284.936	286.214
21	287.649	287.72	286.629	286.382
22	291.858	286.804	285.459	285.743
23	286.187	290.831	289.752	289.036
24	285.962	290.857	289.776	289.314
25	287.551	285.353	284.304	286.041
26	287.856	288.034	286.939	286.849
27	288.199	288.389	287.291	287.211
28	287.445	287.7	286.604	285.868
29	287.645	287.774	286.684	286.474

Supplementary Table 4 – Core transition energies. Core excited states transition energies for the various active spaces considered.

Supplementary Methods 1 - Theoretical calculations

A. Vibrational mode frequencies:

The low-frequency vibrational spectrum was simulated using Gaussian 03, a program suite[1] with unrestricted density functional theory (DFT). The nonlocal B3LYP functional hybrid method was employed and the standard 6-31G(d) basis set was used for the geometry optimization and vibrational energy analysis.

Table S2 shows the calculated mode frequencies for the dimer. Some of these modes have an intramolecular character, as shown in the table by comparison with the calculated modes for the monomer reported by Lazarevic *et al.*[2] There are also degenerate inter/intramolecular modes as discussed in the main article.

B. Optical absorption spectrum

Supplementary Fig. 3 shows the experimental ultraviolet (UV) absorption spectrum of IBP[3,4] in ethanol. It exhibits a structured band with a maximum at ~4.7 eV (262 nm).

The UV spectrum was calculated using multi-configurational self-consistent field (MCSCF) calculations at the cc-pVDZ/CASSCF(6/6) level of theory with the MOLPRO package.[5–12] This valence-excited state calculation was used as a starting point of the subsequent core-excited state RASSCF calculation discussed in the next section. It is compared to the experimental spectrum in Supplementary Fig. 3. In order to match the experimental spectrum, a shift of 3.5 eV was applied to the calculated transition energies and the resulting agreement is satisfactory.[13,14] The shift originates from basis set incompleteness, active space size limitation and relativistic effects.[14] It can be reduced by using second-order perturbation theory CASPT2 and RASPT2.[15] In the MCSCF approach, each eigenstate is represented by a sum of determinants, each corresponding to a configuration of different occupied and unoccupied orbitals. The main configurations contributing to each state and their respective coefficients are highlighted in Table S3, while for a visualization of the orbital shape, refer to Supplementary Fig. 4. Each orbital within the configuration is given a label representing its occupation (2 for double occupancy, 0 for unoccupied orbitals and a and b for single occupancy with spin up and down respectively). For the cc-pVDZ/RASSCF(6/6) level of theory chosen, the considered six orbitals range from HOMO-2 to LUMO+2. As seen in Table S3, the 273 nm (4.542 eV) transition is dominated by HOMO → LUMO+1 and HOMO - 1 → LUMO excited determinants, the 266 nm (4.66 eV) one by HOMO → LUMO, HOMO-1 → LUMO+1, HOMO-2 → LUMO and HOMO → LUMO+2 singly excited determinant, and finally a HOMO → LUMO doubly excited determinant. The 263 nm (4.714 eV) transition is composed of HOMO-2 → LUMO+1 and HOMO-1→LUMO+2 singly excited determinant, and (HOMO/HOMO-1 → LUMO+1), (HOMO-1→LUMO/LUMO+1) doubly excited determinants. For a full description of the transition character, refer to Table S3.

C. Carbon K-edge absorption spectrum

To calculate the C K-edge absorption spectrum, we used restricted active space core-excited states calculations (RASSCF)[16,17] for each carbon deepest core molecular orbitals (MO). This

multireference method has been used successfully to compute molecular core-excited states[18–20]. The Douglas-Kroll-Hess Hamiltonian at the second order was used to account for relativistic corrections important for core excited state computation.

The carbon core MOs are well approximated by localized 1s atomic orbitals (AO) in most cases and we thus label these MOs by the label of the associated carbon. The MOs are represented in Supplementary Fig. 5 where we have chosen a small isovalue (±0.03) contour to highlight the influence of the local environment. As can be seen for the carbon atoms in the ring, this approximation breaks down for nearby carbon atoms possessing a similar environment. The nearby 1s AOs typically get hybridized into a bonding/antibonding pair as can be seen for MOs 19 and 20. As a consequence, the transition energies and oscillator strength for these different MOs exhibit different values although their local environment is similar.

For each carbon atom, the computation was achieved by first rotating the carbon core MO into the active space (AS), freezing it to double occupancy without re-optimization and carrying out a RASSCF calculation. This step ensures that the transition matrix elements with the subsequently calculated core excited states are computed within the same active space labelling. Then, the core orbital is restricted to single occupancy and the two lowest lying core excited states are computed. Finally, transition electric dipole matrix elements are computed between ground, valence and core excited states. The CASSCF computation of valence-excited state discussed in the previous section was used as a starting set of orbitals for the RASSCF one to ensure the computation of transition matrix elements between similar active spaces and to speed up convergence.

In order to ensure the suitability of the chosen active space, the RASSCF calculations were repeated for different active space sizes until convergence was reached. Supplementary Fig. 6 shows the stick spectra calculated at the cc-pVDZ/RASSCF(7/6), cc-pVDZ/RASSCF(7/7), cc-pVDZ/RASSCF(9/8) and cc-pVDZ/RASSCF(7/8) labelled AS1, AS2, AS3 and AS4 respectively. Each of these active spaces include the relevant carbon core orbital and the following orbitals:

- Cn 1s, HOMO-2, HOMO – 1, HOMO, LUMO and LUMO+1 for AS1,
- Cn 1s, HOMO-2, HOMO – 1, HOMO, LUMO, LUMO+1 and LUMO+2 for AS2,
- Cn 1s, HOMO-3, HOMO – 2, HOMO – 1, HOMO, LUMO, LUMO+1 and LUMO+2 for AS3,
- Cn 1s, HOMO-2, HOMO – 1, HOMO, LUMO, LUMO+1, LUMO+2 and LUMO+3 for AS4.

Where Cn 1s indicates the 1s orbital of the nth atom that has been translated into the active space and restricted to single occupancy.

Supplementary Fig. 6 displays the stick XAS spectra for each of the four active space compared with the experimentally measured ones. The ab initio computation provides naturally transition energies matching the observed pre-edge and C K-edge energies.

Supplementary Fig. 7 shows sticks diagrams of the transition energies values (top) and of the transition dipole moments norms (bottom). The active space AS1 and AS4 give significantly different results compared to the other two computations. The transition energies in eV are summarized in Table S4 for the four calculations. We thus consider that convergence is reached

for the other ones. Our interpretation in the main text is based on the AS3 computation because this computation is consistent with AS2 while being a larger active space and provides a better agreement with the measured XAS and CD spectra. Strength of the transitions (sticks of Supplementary Fig.s 6, 7 and 3a) are calculated the electric and magnetic transition dipole moments given by $\boldsymbol{\mu}_{cg} = \mathrm{e} < \varphi_c\,|\mathbf{r}|\,\varphi_g >$ and $\mathbf{m}_{cg} = \frac{e}{2mc} < \varphi_c\,|\mathbf{r} \times \mathbf{p}|\,\varphi_g >$ respectively where φ_c and φ_g are the core-excited and ground many-body states. Low frequency oscillations are expected to significantly change the geometry of the molecule and consequently the value of the transitions by altering the projections. On the contrary, high frequency oscillations are generally connected to smaller amplitudes and thus smaller change in the projections which may remain hidden in the experimental noise.

The CD stick spectra were calculated using the following sum-over-states expression:[21]

$$\mathrm{CD} = \sum_{c} \mathrm{Im}(\boldsymbol{\mu}_{cg} \cdot \mathbf{m}_{cg})\ \delta(\omega - \omega_{cg})$$

Where $\boldsymbol{\mu}_{cg}$ and $\mathbf{m}_{cg}$ are the transition electric and magnetic dipoles and ω_{cg} are the transition energies. $\mathrm{R}_{cg} = \mathrm{Im}(\boldsymbol{\mu}_{cg} \cdot \mathbf{m}_{cg})$ is also known as the rotatory strength. Phenomenological broadening can also be introduced by replacing the Dirac distribution $\delta(\omega - \omega_{cg})$ by Lorentzian functions. The phenomenological broadening of the carbon core-excited states is inversely proportional to their lifetimes. The literature provides highly varying values ranging from the attosecond to the few tens of femtoseconds regime. We thus have chosen to display only unbroadened stick spectra.

Supplementary Methods 2- Steady-state optical spectroscopy

A. Raman spectroscopy

Although already reported in the literature[22], we recorded the low-frequency Raman scattering of the IBP powder (Supplementary Fig. 9). The micro-Raman apparatus used to acquire the spectra at room temperature in backscattering geometry, under cross-polarization, consist in a triple-monochromator spectrometer (Horiba Jobin Yvon, model T64000) set in double-subtractive/single configuration and equipped with 1800 grooves/mm gratings. The Raman spectra were excited by a 647.1 nm argon/krypton ion laser and detected using a CCD camera (256x1024 pixels) cryogenically cooled by liquid nitrogen. The incident radiation was focused onto the sample surface with a spot size of about 1 μm^2 through an 80X objective having a NA = 0.75. The calibration of the frequency shift was obtained using the emission lines[23] of an Argon lamp. In this configuration, the set-up resolution was about 0.36 cm^{-1}/pixel over the scanned spectral range.

B. FTIR measurements

We also recorded the IR-absorption spectrum of the sample, shown in Supplementary Fig. 10. We performed FTIR absorption measurements of a 500 μm thick pellet of pure IBP powder (Sigma Aldrich), prepared using a standard hand press. The IBP THz transmission was recorded between 0 and 150 cm^{-1}, with a spectral resolution of 0.5 cm^{-1}. Data were acquired in sample compartment,

under vacuum conditions. A vertex70v (Bruker) spectrometer was used, equipped with a thermal source (Globar) and a He-cooled Bolometer detector. The transmission spectrum was obtained by averaging 20 measurements, acquired with 128 scans at 2.5 kHz detector scanning rate. A Blackman-Harris (3-term) apodization filter was used in the Fourier transform. The same procedure was employed to measure the source spectrum only and calculate the sample absorbance. To avoid detector saturation a pinhole of 2.5 mm was used to geometrically block a fraction of the beam.

C. Optical Photoluminescence spectrum

The steady-state photoluminescence (PL) spectrum of the IBP powder is shown in Supplementary Fig. 10 and it agrees with the literature.[24] It shows a structured band which is strongly Stokes shifted with respect to the absorption band maximum, such that any low frequency Raman modes are not affected by the presence of the PL. The PL spectrum was obtained exciting a IBP powder sample with UV radiation at 262 nm (4.733 eV) from the synchrotron source Elettra (BL10.2-IUVS beamline, Trieste, Italy). The re-emitted light from the sample was collected in backscattering geometry using a Czerny-Turner spectrometer (Trivista 557, Princeton Instruments). The radiation power on the sample was ~7 μW.

Supplementary Methods 3 - Ultrafast optical spectroscopy

In order to verify the occurrence of ground state vibrational coherences generated by the impulsive stimulated Raman scattering, we also carried out femtosecond experiments using a deep-ultraviolet (UV) broadband continuum (270-350 nm) as probe, in order to monitor the response of the first absorption band of IBP below 300 nm (Supplementary Fig. 3). The hypothesis is that coherent ground state vibrations could be detected via a temporal modulation of the absorption spectrum, as reported in ref. [25]. We used the 20 kHz set-up described in ref. [26,27], and excited the system using the third harmonic generation to reproduce the conditions of the X-ray experiment. The pump fluence was ~ 0.6 mJ/cm^2 and the pulse duration of ~130 fs, largely sufficient to generate low-frequency modes by ISRS. We performed transient absorption (TA) experiments on both IBP dissolved in ethanol and IBP in powder form deposited on a quartz substrate.

Supplementary Fig. 12 shows the time-wavelength plots of the TA of IBP in solution and Supplementary Fig. 13 shows the TA spectra at specific time delays. It can be seen that the entire spectral range is dominated by excited state absorption (positive signals) with the 260-310 nm region decaying and the 310-360 nm region growing on a commensurate time scale, and stabilising over long times. This suggests the decay of a singlet state into a long-lived, presumably, triplet state. Most importantly for the present purposes, the data does not show evidence of coherent oscillations. This could be due to several reasons that could all concur: a) they do not affect the ground state absorption since the latter is due to a valence excitation that is delocalised over the entire molecular edifice; b) the excited state absorption, which overwhelms the signal, may shade any effects due to the weaker ground state absorption and therefore hinder their observation; c) they are specific to the powder form, as they are intermolecular modes.

We therefore also attempted the experiment on the powder samples deposited on a quartz suprasil (UV transparent) substrate, but could not observe any signal. There are several reasons for this: a) the experiment was performed at 20 kHz, which means that the same spot is repeatedly excited by the pump pulse and therefore sample damage occurs, even though the sample is moved during the data acquisition. Indeed, sample damage could be detected by eye; b) the IBP powder is highly scattering in the deep-UV; c) sample inhomogeneities are such that moving the sample introduces a significant noise, let alone the sample damage itself.

In summary, we cannot conclude from the deep-UV TA spectra whether low-frequency modes of IBP are impulsively generated by 266 nm excitation in solution or in powder form. Remarkably the lower repetition rate of the FEL experiment, combined with a probe, tuned to core transitions, which is less prone to surface scattering seems to yield a superior sensitivity shown in this experiment, highlighting dynamics hardly detectable in laser labs.

Supplementary References

1. Frisch, M. J. *et al.* Gaussian 03, Revision C.03.
2. Lazarević, J. J. *et al.* Intermolecular and low-frequency intramolecular Raman scattering study of racemic ibuprofen. *Spectrochim. Acta. A. Mol. Biomol. Spectrosc.* **126**, 301–305 (2014).
3. Ma, M. Y., Zhu, Y. J., Li, L. & Cao, S. W. Nanostructured porous hollow ellipsoidal capsules of hydroxyapatite and calcium silicate: Preparation and application in drug delivery. *J. Mater. Chem.* **18**, 2722–2727 (2008).
4. Louden, D. *et al.* Flow injection spectroscopic analysis of model drugs using on-line UV-diode array, FT-infrared and 1H-nuclear magnetic resonance spectroscopy and time-of-flight mass spectrometry. *Analyst* **125**, 927–931 (2000).
5. Amos, R. D., Andrews, J. S., Handy, N. C. & Knowles, P. J. Open Shell {M{ø}ller--Plesset} Perturbation Theory. *Chem Phys Lett.* **185**, 256–264 (1991).
6. Knowles, P. J., Andrews, J. S., Amos, R. D., Handy, N. C. & Pople, J. A. Restricted {Møller--Plesset} Theory for Open Shell Molecules. *Chem Phys Lett.* **186**, 130–136 (1991).
7. Knowles, P. J., Hampel, C. & Werner, H.-J. Coupled Cluster Theory for High Spin Open Shell Reference Wavefunctions. *J Chem Phys* **99**, 5219–5227 (1993).
8. Knowles, P. J. & Handy, N. C. A New Determinant-based Full Configuration Interaction Method. *Chem Phys Lett.* **111**, 315–321 (1984).
9. Knowles, P. J. & Handy, N. C. A Determinant Based Full Configuration Interaction Program. **54**, 75–83 (1989).
10. Knowles, P. J. & Werner, H.-J. An Efficient Second Order {MCSCF} Method for Long Configuration Expansions. *Chem Phys Lett.* **115**, 259–267 (1985).
11. Knowles, P. J. & Werner, H.-J. An Efficient Method for the Evaluation of Coupling Coefficients in Configuration Interaction Calculations. *Chem Phys Lett.* **145**, 514–522 (1988).

12. Knowles, P. J. & Werner, H.-J. Internally Contracted Multiconfiguration Reference Configuration Interaction Calculations for Excited States. **84**, 95–103 (1992).

13. Scholz, M. *et al.* Core hole-electron correlation in coherently coupled molecules. *Phys. Rev. Lett.* **111**, 1–5 (2013).

14. Norman, P. & Dreuw, A. Simulating X-ray Spectroscopies and Calculating Core-Excited States of Molecules. *Chem. Rev.* **118**, 7208–7248 (2018).

15. Sauri, V. *et al.* Multiconfigurational second-order perturbation theory restricted active space (RASPT2) method for electronic excited states: A benchmark study. *J. Chem. Theory Comput.* **7**, 153–168 (2011).

16. Malmqvist, P. Aake., Rendell, A. & Roos, B. O. The restricted active space self-consistent-field method, implemented with a split graph unitary group approach. *J. Phys. Chem.* **94**, 5477–5482 (1990).

17. Coe, J. P. & Paterson, M. J. Multireference X-ray emission and absorption spectroscopy calculations from Monte Carlo configuration interaction. *Theor. Chem. Acc.* **134**, 58 (2015).

18. Guo, M. *et al.* Molecular Orbital Simulations of Metal 1s2p Resonant Inelastic X-ray Scattering. *J. Phys. Chem. A* **120**, 5848–5855 (2016).

19. Alagia, M. *et al.* The soft X-ray absorption spectrum of the allyl free radical. *Phys Chem Chem Phys* **15**, 1310–1318 (2013).

20. Pinjari, R. V., Delcey, M. G., Guo, M., Odelius, M. & Lundberg, M. Restricted active space calculations of L-edge X-ray absorption spectra: From molecular orbitals to multiplet states. *J. Chem. Phys.* **141**, 124116 (2014).

21. Berova, N., Nakanishi, K. & Woody, R. *Circular dichroism : principles and applications*. (Wiley-VCH, 2000).

22. Lazarević, J. J. *et al.* Intermolecular and low-frequency intramolecular Raman scattering study of racemic ibuprofen. *Spectrochim. Acta. A. Mol. Biomol. Spectrosc.* **126**, 301–305 (2014).

23. NIST Atomic Spectra Database Lines Data. https://physics.nist.gov/cgi-bin/ASD/lines1.pl?spectra=Ar+&limits_type=0&low_w=488&upp_w=490&unit=1&submit=Retrieve+Data&de=0&format=0&line_out=0&en_unit=0&output=0&bibrefs=1&page_size=15&show_obs_wl=1&show_calc_wl=1&unc_out=1&order_out=0&max_low_enrg=&sh.

24. Leising, G. *et al.* Physical aspects of dexibuprofen and racemic ibuprofen. *J. Clin. Pharmacol.* **36**, 3s–6s (1996).

25. van der Veen, R. M., Cannizzo, A., van Mourik, F., Vlček, A. & Chergui, M. Vibrational Relaxation and Intersystem Crossing of Binuclear Metal Complexes in Solution. *J. Am. Chem. Soc.* **133**, 305–315 (2011).

26. Auböck, G. *et al.* Femtosecond pump/supercontinuum-probe setup with 20 kHz repetition rate. *Rev. Sci. Instrum.* **83**, 093105 (2012).

27. Auböck, G., Consani, C., Mourik, F. van & Chergui, M. Ultrabroadband femtosecond two-dimensional ultraviolet transient absorption. *Opt. Lett.* **37**, 2337–2339 (2012).

28. Szabo, A. & Ostlund, N. S. *Modern Quantum Chemistry: Introduction to Advanced Electronic Structure Theory*. (Dover Publications, 2012).
