# Supplementary material for: Element- and enantiomer-selective visualization of molecular motion in real-time
Source: Nat Commun. 2023 Jan 24;14:386. doi: 10.1038/s41467-023-36047-5 (PMC9873934; doi:10.1038/s41467-023-36047-5)
Supplement: Supplementary file 2 — Description of Additional Supplementary Files [file 41467_2023_36047_MOESM2_ESM.pdf]

Supplementary Movie 1
Description: Animation of the dimer oscillation at 21.5 cm^{-1}.

Supplementary Movie 2
Description: Animation of the dimer oscillation at 22.6 cm^{-1}.

Supplementary Movie 3
Description: Animation of the dimer oscillation at 28.6 cm^{-1}.
