# Supplementary figures and images for: Element- and enantiomer-selective visualization of molecular motion in real-time
Source: Nat Commun. 2023 Jan 24;14:386. doi: 10.1038/s41467-023-36047-5 (PMC9873934; doi:10.1038/s41467-023-36047-5)

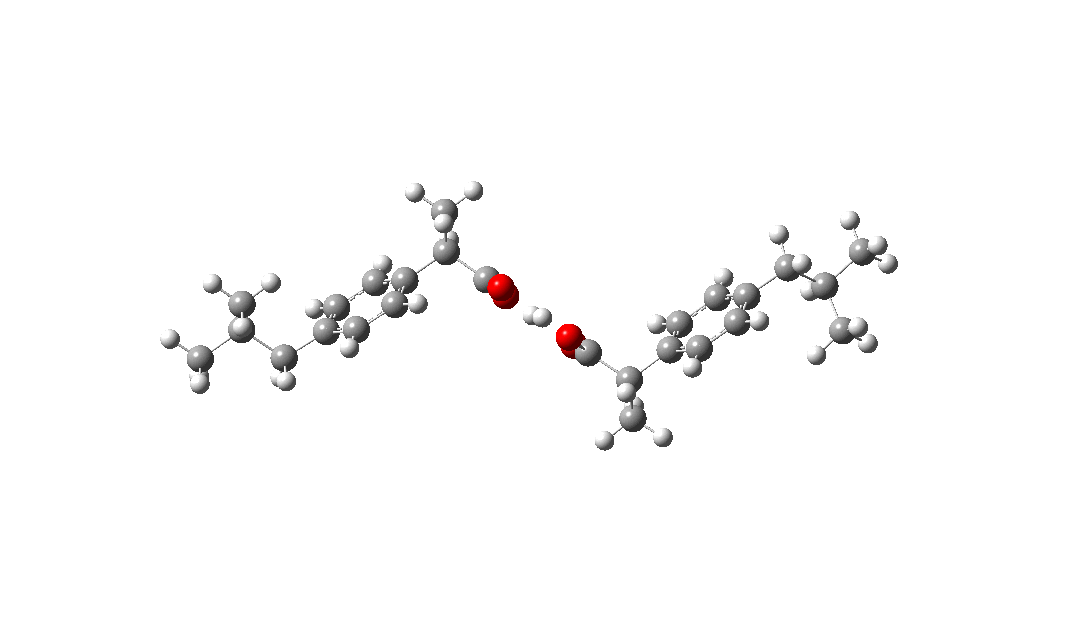

Supplement: Supplementary file 3 — Supplementary Movie 1 [file 41467_2023_36047_MOESM3_ESM.gif]

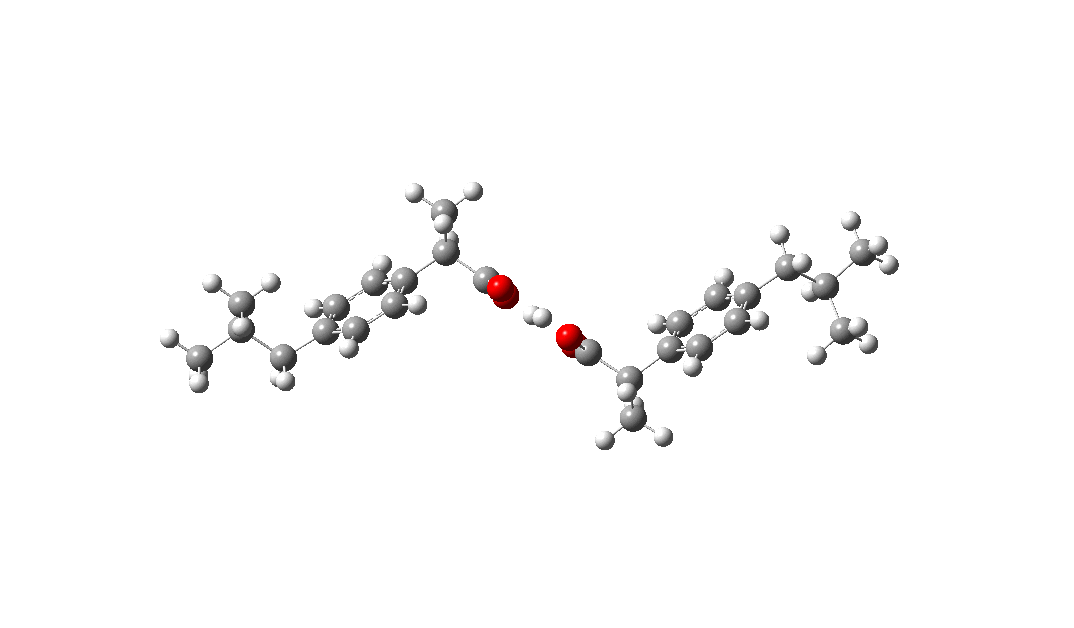

Supplement: Supplementary file 4 — Supplementary Movie 2 [file 41467_2023_36047_MOESM4_ESM.gif]

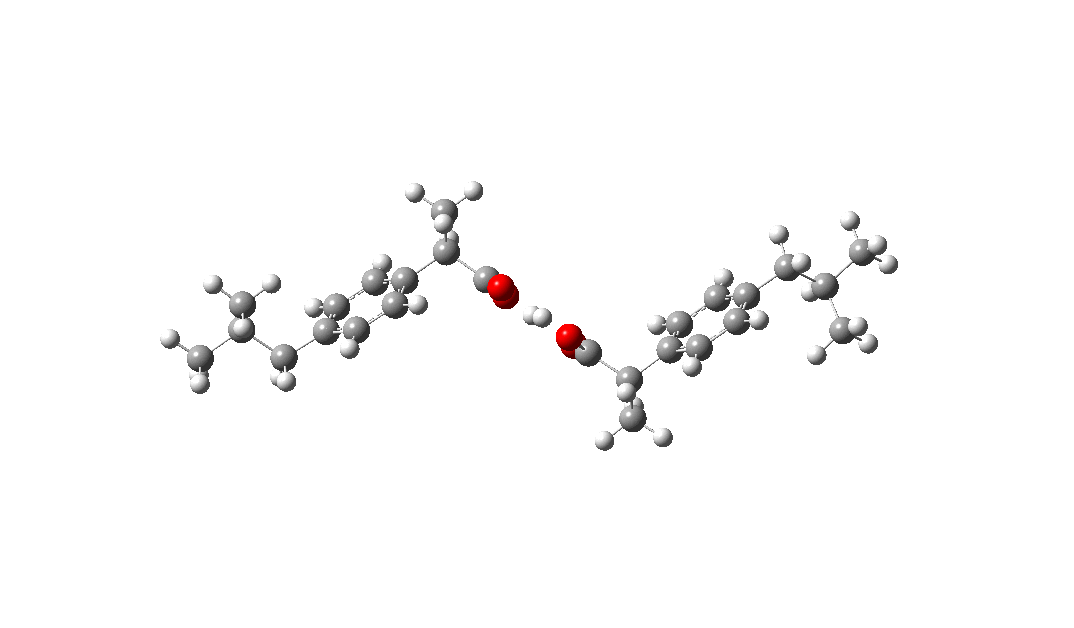

Supplement: Supplementary file 5 — Supplementary Movie 3 [file 41467_2023_36047_MOESM5_ESM.gif]
